# Occurrence and Monitoring of the Zoonotic Pathogen *Yersinia pseudotuberculosis* in Various Zoo Animal Species

**DOI:** 10.3390/microorganisms13030516

**Published:** 2025-02-26

**Authors:** Lara Luisa Riede, Tobias Knauf-Witzens, Uta Westerhüs, Rebecca Bonke, Karen Schlez, Kathrin Büttner, Jörg Rau, Dominik Fischer, Lisa Grund, Marco Roller, Andreas Frei, Stefan Hertwig, Jens Andre Hammerl, Claudia Jäckel, Christine Osmann, Martin Peters, Reinhard Sting, Tobias Eisenberg

**Affiliations:** 1 Zoological-Botanical Garden Wilhelma, 70376 Stuttgart, Germany; lara.riede@wilhelma.de (L.L.R.);; 2 Opel Hessian Zoo Foundation, 61476 Kronberg/Taunus, Germany; 3 Hessian State Laboratory, 35392 Giessen, Germanytobias.eisenberg@lhl.hessen.de (T.E.); 4 Unit for Biomathematics and Data Processing, Justus Liebig University Giessen, 35392 Giessen, Germany; 5 Chemical and Veterinary Analysis Agency Stuttgart, 70736 Fellbach, Germany; 6 Zoo Wuppertal, 42117 Wuppertal, Germany; 7 Zoo Karlsruhe, 76137 Karlsruhe, Germany; 8 Walsrode World Bird Park, 29699 Walsrode, Germany; 9 Department Biological Safety, German Federal Institute for Risk Assessment, 10589 Berlin, Germanyclaudia.jaeckel@bfr.bund.de (C.J.); 10 Zoo Dortmund, 44225 Dortmund, Germany; 11 Chemical and Veterinary Investigations Office Westphalia, 59821 Arnsberg, Germany; martin.peters@cvua-westfalen.de; 12 Institute of Hygiene and Infectious Diseases of Animals, Justus Liebig University Giessen, 35392 Giessen, Germany

**Keywords:** *Yersinia pseudotuberculosis*, zoonosis, mammals and birds, prevalence, clinical and pathological signs, prevention, seasonal occurrence, antimicrobial testing

## Abstract

Infections with the zoonotic pathogen *Yersinia* (*Y*.) *pseudotuberculosis* are commonly causing clinical diseases and acute deaths in various mammal and bird species in zoos. These findings prompted us to conduct a targeted study that included animals of 18 German and 1 Swiss zoo that had been affected by the pathogen previously. This study provides a comprehensive overview of susceptible zoo animal species, clinical signs, therapies, antimicrobial resistance, pathogen monitoring, and prophylactic measures. In addition, fecal samples from species with documented infections and organ samples from deceased mammals and birds from two of the participating zoos, the urban zoo Wilhelma and the rural Opel Zoo, were examined for *Y. pseudotuberculosis* using both direct plating and enrichment. The overall prevalence of *Y. pseudotuberculosis* was 3.1% at the Opel Zoo and 1.3% at the Wilhelma. Fecal samples yielded positive results in 1.4% of the tested samples from the Opel Zoo but none from the Wilhelma. Among carcasses, 16.7% and 1.7% tested positive at the Opel Zoo and the Wilhelma, respectively (*p* = 0.006). *Y. pseudotuberculosis* was significantly more frequently isolated during the cold season (*p* = 0.002). Affected animals often died suddenly, displaying no or only non-specific clinical signs, but postmortem examinations revealed septicemia with multiple bacterial organ abscesses. Rodents, ruminants, primates, and Piciformes were the most commonly affected orders. Considering the zoonotic potential of *Y. pseudotuberculosis*, this research underscores the importance of investigations in zoos in accordance with the targets of the One Health approach.

## 1. Introduction

*Yersinia* (*Y.*) *pseudotuberculosis* belongs to the family *Yersiniaceae* and is an important zoonotic pathogen that occurs worldwide [1,2]. Along with *Y. enterocolitica* and *Y. pestis*, *Y. pseudotuberculosis* is one of the three *Yersinia* species that are pathogenic to both humans and animals [3]. Rodents, game animals, and birds, especially free-living species, are a relevant reservoir for *Y. pseudotuberculosis* [2,4,5]. Wild rodents such as rats and mice are often asymptomatic carriers and shedders of this pathogen and are regarded as a fundamental link of the infection chains [6,7]. *Y. pseudotuberculosis* infections are commonly known as pseudotuberculosis and especially in rodents as rodentiosis.

In contrast to *Y. pseudotuberculosis*, pigs are the main reservoir of *Y. enterocolitica*. Although other species, including many zoo animals, can be infected with *Y. enterocolitica*, the infection is usually limited to the intestine and the course is usually asymptomatic and rarely lethal [8,9]. A further important pathogen is *Y. pestis*, which is closely related to *Y. pseudotuberculosis*. In endemic areas, a large number of animals species is susceptible, however, rodents are a well-studied reservoirs of *Y. pestis*, and they are usually asymptomatically affected. In contrast to other *Yersinia* spp., *Y. pestis* is transmitted by bites from infected arthropods or by droplet infection and characterized by endemic occurrence [10]. Infections with *Y. pseudotuberculosis* typically occur via the oral route and usually cause self-limiting gastrointestinal infections in humans and animals [11,12]. More rarely, symptoms such as “pseudoappendicitis” or severe illnesses in the form of enteritis, mesenteric lymphadenitis, fever, and, in rare cases, septicemia occur in humans [2,13]. On average, 2700 cases of human yersiniosis caused by *Y. enterocolitica* and *Y. pseudotuberculosis* are reported in Germany every year. However, the majority of these cases are caused by *Y. enterocolitica* [14,15]. In Germany, since the amendment of the Infection Protection Act (IfSG) in 2017, there has been a reporting obligation for proven cases of *Y. enterocolitica* as well as for *Y. pseudotuberculosis* in humans.

In animals, *Y. pseudotuberculosis* has a broad host range and has been detected in many different mammals and birds [16]. In most cases, these animals are asymptomatic carriers, and diseases emerge sporadically. However, outbreaks with high mortality rates were likewise described, especially among wild hares and zoo animals [16,17,18].

In zoos, non-human primates, several antelope species, rodents such as capybaras as well as various birds, especially representatives of the order Piciformes, are very sensitive to *Y. pseudotuberculosis* infections [2,7]. Acute pseudotuberculosis manifests as fulminant septicemia, often associated with sudden deaths or deaths after one to three days [19]. Some reports describe the isolation of *Y. pseudotuberculosis* from birds and mammals kept in zoos around the world that suddenly died from this infection [20]. In many of those animals, hepatitis, splenitis, and lymphadenitis of the mesenteric lymph nodes with miliary necrotic foci were observed during postmortem examinations [21,22].

This study analyzes the occurrence and relationships of the zoonotic pathogen *Y. pseudotuberculosis* in zoo animals living in an urban and a rural zoo in Germany. The prevalence and infectious sites of *Y. pseudotuberculosis* were determined by sampling of live and dead zoo animals and subsequent direct bacterial culture and *Yersinia* enrichment in both zoos as well as for wild small mammals and wild birds frequenting at the Wilhelma zoo. The results are intended to help assess risk factors and develop targeted biosecurity measures to prevent infections in animals and humans. In addition, retrospective clinical cases from other zoos, mainly from 18 German zoos and one Swiss zoo, as well as some case reports from the literature, are presented. This study provides an overview of the range of susceptible animal species, clinical signs, and postmortem findings in affected animals and the susceptibility patterns of the isolates obtained.

## 2. Materials and Methods

### 2.1. Sample Collection at the Wilhelma Zoo and the Opel Zoo

The occurrence of *Y. pseudotuberculosis* was examined in the urban Zoological-Botanical Garden Wilhelma in Stuttgart (Wilhelma) in the federal state of Baden-Wuerttemberg (Germany) and the rural Opel Zoo in Kronberg/Taunus (Opel Zoo) in the federal state of Hesse (Germany) over a period of twelve months from the beginning of June 2023 to the end of May 2024. Only samples collected at these two zoos were used for monitoring. The focus was on examinations of organ samples from deceased zoo animals from the Wilhelma and the Opel Zoo (vertebrates—except reptiles, amphibians, and fish), which were examined as part of the gross pathology at the Chemical and Veterinary Analysis Agency Stuttgart (CVUAS), Fellbach (Germany), and the Hessian State Laboratory (LHL), Giessen (Germany). Fecal samples from zoo animal species, in which clinical cases of *Y. pseudotuberculosis* infections had occurred, and fecal samples from animals with suspicious clinical signs were analyzed. In this context, mammals and birds that were tested positive in enclosures at the Wilhelma Zoo and the Opel Zoo within the past five years are referred to as ‘hotspot species’. At the Wilhelma, all wild small mammals, especially pest rodents, and birds found dead on the site of the Wilhelma were also examined in the in-house postmortem room.

The ‘hotspot species’ at the Wilhelma included the animal species bonobo (*Pan paniscus*), squirrel monkey (*Saimiri sciureus*), Goeldi’s marmoset (*Callimico goeldii*), maned wolf (*Chrysocyon brachyurus*), short-eared elephant shrew (*Macroscelides proboscideus*), pearl-necked dove (*Spilopelia chinensis*), red-rumped parrot (*Psephotus haematonotus*), serval (*Leptailurus serval*), and Seba’s short-tailed bats (*Carollia perspicillata*). *Y. pseudotuberculosis* could not be detected in previous preliminary investigations by cultivation in servals and Seba’s short-tailed bats, but the samples were positive for the *ail* gene by PCR. Therefore, these two animal species were included in the monthly sampling, as *Y. pseudotuberculosis* is known to occur particularly in Seba’s short-tailed bats [23].

The ‘hotspot species’ at the Opel Zoo included the species bush hyrax *(Heterohyrax brucei*), African pygmy goat (*Capra aegagrus hircus*), impala (*Aepyceros melampus*), blackbuck (*Antilope cervicapra*), and emperor tamarin (*Saguinus imperator*).

In addition to the zoo animals, small mammals and wild birds that had been found dead on the grounds of the Wilhelma Zoo were also examined by culture for *Y. pseudotuberculosis* using pooled intestinal and liver samples per animal. These included the animal species house mouse (*Mus musculus*), brown rat (*Rattus norvegicus*), Eurasian red squirrel (*Sciurus vulgaris*), brown hare (*Lepus europaeus*), dormouse (*Glis glis*), red fox (*Vulpes vulpes*), least weasel (*Mustela nivalis*), hedgehog (*Erinaceus europaeus*), gray heron (*Ardea cinerea*), sparrow (*Passer domesticus* and *P. montanus*), blue tit (*Cyanistes caeruleus*), domestic pigeon (*Columba livia domestica*), wood pigeon (*Columba palumbus*), green woodpecker (*Picus viridis*), common buzzard (*Buteo buteo*), mallard (*Anas platyrhynchos*), moorhen (*Gallinula chloropus*), and Egyptian goose (*Alopochen aegyptiacus*).

Furthermore, one-time fecal samples were collected from clinically healthy animals kept at the Wilhelma Zoo and the Opel Zoo, which were exposed to an increased risk of infection. These included animals in the same enclosure as ‘hotspot species’ animals in enclosures with high rodent infestation or species known to be very sensitive to *Y. pseudotuberculosis* infection, such as non-human primates.

### 2.2. Microbiological Examination of the Samples

Pooled liver and intestinal samples from all deceased zoo animals from both zoos, as well as small mammals and birds from the Wilhelma Zoo that died on the grounds, were used for bacteriological examinations. All feces and organ samples were streaked directly in a three-loop smear on CIN *Yersinia* selective agar according to Schiemann (CIN-Cefsulodin-Irgasan^TM^-Novobiocin agar; ThermoFisher Scientific, Darmstadt, Germany) followed by incubation for 24 ± 2 h at 30 ± 1 °C and then further for 24 ± 2 h at room temperature. For enrichment of *Yersinia*, 1 g of each sample was transferred to 9 mL of phosphate buffered peptone broth supplemented with 1% mannitol and 0.15% bile salt (PBSMBB; ThermoFisher Scientific, Darmstadt, Germany) and homogenized for 1 min by mechanical mixing. The PBSMSB enrichment was incubated aerobically at 25 ± 1 °C for 48 ± 4 h. Subsequently, 0.5 mL of the incubated enrichment broth was transferred to 4.5 mL of sterile 0.5% KOH (potassium hydroxide; ThermoFisher Scientific, Darmstadt, Germany) solution and mixed well with a pipette. After 20 ± 5 s, 100 µL were transferred to a CIN agar plate and incubated at 30 ± 1 °C for 24 ± 2 h and then further for 24 ± 2 h at room temperature.

For biosafety measures, masks and disposable materials like gloves were worn during the collection and examination of clinical samples. In the case of laboratory instruments (scissors, forceps), a separate instrument was used for each sample and then autoclaved before the next use.

After the first and the second incubation of the CIN agar plates, colonies suspected of containing *Y. pseudotuberculosis*, both from direct smear culture on CIN agar plates as well as cultures after enrichment, were pre-tested using MALDI-TOF MS (matrix-assisted laser desorption/ionization time-of-flight mass spectrometry, MALDI Biotyper^®^ sirius system, Bruker Daltonics GmbH, Bremen, Germany). In case of multiple suspicious colonies, one representative colony was checked unless there were macroscopically visible phenotypic differences. Material was applied to two target positions per colony. Cultures of suspicious colonies were subcultured for 24 ± 2 h on Columbia agar with 5% sheep blood (BD, Heidelberg, Germany) and Gassner agar (Merck, Darmstadt, Germany) to obtain pure cultures for definite MALDI-TOF MS analysis.

After preparation of the colonies with the direct transfer protocol, MALDI-TOF mass spectra of the samples were identified using the commercial “research use only” (RUO) MBT_K_ database (revision K, 2022; Bruker Daltonics GmbH, Bremen, Germany) and supplemented with user-made entries from the MALDI user platform MALDI-UP, as described previously [24,25]. The targeted identification of *Y. pseudotuberculosis* had been validated according to the technical guidelines created by the BVL working group MALDI-TOF MS [26]. If an identification score value between 2.0 and 3.0 was achieved for both target positions, the colony was accepted as confirmed *Y. pseudotuberculosis* according to the hints of the manufacturer (Bruker Daltonics GmbH, Bremen, Germany). Successfully retested subcultures were finally preserved by freezing at −80 ± 5 °C on ceramic beads in cryovials (Protect©, Transia, Ober-Mörlen, Germany).

In addition, the isolates were sent to the German Federal Institute for Risk Assessment (BfR) for antimicrobial susceptibility testing.

### 2.3. Antimicrobial Susceptibility Testing

Antimicrobial susceptibility testing of the *Y. pseudotuberculosis* isolates was carried out according to the CLSI protocol for the agar disc diffusion method on Mueller–Hinton (MH) agar (ThermoFisher Scientific, Darmstadt, Germany) incubated at 30 °C for 17 ± 1 h [27]. The antimicrobial discs (ThermoFisher Scientific, Darmstadt, Germany) used contained amoxicillin/clavulanate (AMC: 20/10 μg), amikacin (AK: 30 μg), ampicillin (AMP: 10 μg), cefepime (FEP: 30 μg), cefotaxime (CTX: 30 μg), ceftazidime (TAZ: 30 μg), chloramphenicol (CHL: 30 μg), ciprofloxacin (CIP: 5 μg), erythromycin (E: 15 μg), florfenicol (FFC: 30 μg), gentamicin (CN: 10 μg), imipenem (IMI: 10 μg), meropenem (MER: 10 μg), nalidixic acid (NAL: 30 μg), norfloxacin (NOR: 10 μg), streptomycin (S: 10 μg), tetracycline (TE: 30 μg), trimethoprim (W: 5 μg), and trimethoprim/sulfamethoxazole (SXT: 1.25/23.75 μg). Reference strains were used during testing as quality control (erythromycin discs: *Staphylococcus aureus* ATCC 25923; imipenem/meropenem discs: *Pseudomonas aeruginosa* ATCC 27853; amoxicillin/clavulanate discs: *Escherichia* (*E.*) *coli* ATCC 35218) according to the CLSI guidelines. The quality of the remaining discs was evaluated using *E. coli* ATCC 25922. The evaluation was based on the size of the measured inhibition zones according to the values of the clinical breakpoints for human medicine in the CLSI document M100 [27]. There are no validated clinical breakpoints for erythromycin and florfenicol, which is why the isolates were interpreted and described in comparison to each other. A classification was made into (1) no inhibition zone, (2) inhibition zone ≤ 10 mm for both substances, (3) inhibition zone > 10 mm for erythromycin or inhibition zone 11–24 mm for florfenicol, and (4) inhibition zone ≥ 25 mm for florfenicol.

### 2.4. Statistical Analysis

Statistical analysis of the data obtained in the course of the monitoring study at the Wilhelma Zoo and the Opel Zoo over a period of one year was carried out, together with the Unit for Biomathematics and Data Processing of the Faculty of Veterinary Medicine at the Justus Liebig University in Giessen, using the computer program SAS^®^ 9.4 (Base SAS^®^ 9.4 Procedures Guide: Statistical Procedures, 2nd edition ed., 2013, Statistical Analysis System Institute SAS^®^ Inc., Cary, NC, USA). The data from the monitoring at the Wilhelma Zoo and the Opel Zoo were subjected to statistical evaluations using an exact Pearson chi-squared test. In instances where the total number (n) was insufficient for certain inquiries, a descriptive analysis of the data was conducted.

### 2.5. Collection of Y. pseudotuberculosis Cases from Other Zoos

In addition to the cases from the Wilhelma Zoo and the Opel Zoo, another 18 German zoos, 1 Swiss zoo, and a single case from a private keeping (Roklum, Lower Saxony, Germany) were included in the study. The 117 cases were retrospectively analyzed based on pathological findings and records of the course of the disease. The period under review spanned from 2007 to 2024. The data on external cases of *Y. pseudotuberculosis* were kindly provided by the German zoos in Berlin, Cottbus, Donnersberg, Dortmund, Duisburg, Erfurt, Frankfurt, Hamm, Heidelberg, Hodenhagen, Karlsruhe, Munich, Neuwied, Nuremberg, Schwerin, Walsrode, Wuppertal, the German Primate Center Göttingen, from an unnamed Swiss zoo and a private keeping in Lower Saxony upon request. A total of 138 additional *Y. pseudotuberculosis* isolates from wild and domestic mammals from the culture collection of the LHL Giessen and the CVUA Stuttgart were available from cryopreserved stocks and were included in the present study. These isolates from the culture collection were included for a number of investigations, particularly genome comparisons intended to be carried in future. In addition, zoo isolates were compared with non-zoo isolates. The focus of the evaluations was on affected animal species in zoos, the season in which the disease occurred, preliminary reports, the course of the disease, pathomorphological findings, and the *Y. pseudotuberculosis* isolates obtained from organs.

Both, the 132 isolates from the Wilhelma, the Opel Zoo, and external zoos as well as the 138 cryopreserved *Y. pseudotuberculosis* isolates from culture collections are going to be used for genome comparison in a further study. All 270 *Y. pseudotuberculosis* isolates were tested for antimicrobial susceptibility at the BfR in Berlin and were listed on the MALDI-UP catalogue for tracking MALDI-TOF mass spectra (https://maldi-up.ua-bw.de [accessed on 19 February 2025]).

## 3. Results

### 3.1. Data from the Monitoring of the Wilhelma and the Opel Zoo

#### Descriptive and Statistical Analysis

During the study period, a total of 772 samples were tested for *Y. pseudotuberculosis* at the Wilhelma Zoo (n = 613) and the Opel Zoo (n = 159) (Table 1).

From the 613 samples that originated from the Wilhelma Zoo, eight tested positive for *Y. pseudotuberculosis*. Two positive samples came from organs of deceased zoo animals, a red-rumped parrot (*Psephotus haematonotus*) and an eastern rosella (*Platycercus eximius*), which were found dead in the same aviary without prior clinical signs within five days. The red-rumped parrot was a ‘hotspot species’ that had shared an outdoor free-flight aviary with the eastern rosella and several other species of parakeets and cockatoos. The aviaries were accessible for wild birds and pest rodents. The six other positive samples came from wild small mammals (a brown hare, a Eurasian red squirrel, and four brown rats) that were found dead at the Wilhelma. One of the rats and the brown hare were found in the department from which two dead parakeets originated. The other wild small mammals originated from other enclosures of ‘hotspot species’ where cases of pseudotuberculosis had occurred in the last seven years. However, the four rats which were obtained as a part of the regular rodent control in this department showed no abnormalities during postmortem examinations. Two of the animals were juveniles. The Eurasian red squirrel was hit by a slow-moving vehicle on a service road between the outdoor free-flight aviary and the zoo’s depot, but it did not show any macroscopic organ changes.

In December 2023, a fecal sample from a healthy Mesopotamian fallow deer (*Dama mesopotamica*) tested positive for *Y. pseudotuberculosis* as part of a transport investigation at the Opel Zoo. Additionally, the impalas’ (‘hotspot species’) fecal samples were positive in December 2023. At the end of December, a juvenile impala (*Aepyceros melampus*) died due to a *Y. pseudotuberculosis* infection. At the beginning of January 2024, another young impala from the same enclosure and a young lesser flamingo (*Phoeniconaias minor*) died suddenly from pseudotuberculosis. In the postmortem examination, the two impalas showed acute catarrhal enteritis and purulent-necrotizing lymphadenitis of the mesenteric lymph nodes. The lesser flamingo had multiple miliary abscesses in the liver, reproductive organs, and serous membranes. All three animals were cachectic. All other fecal samples, including those from January 2024, tested negative. In total, four animals and one collective fecal sample were found positive for *Y. pseudotuberculosis* at the Opel Zoo (Table 1).

Overall, based on the total number of 772 samples examined, the prevalence of *Y. pseudotuberculosis* was 1.7% (n = 13). At the Wilhelma Zoo, the prevalence from the 613 samples was 1.3% (n = 8), and at the Opel Zoo, the prevalence from the 159 samples was 3.1% (n = 5). No significant difference in the prevalence was found between the two zoos (*p* = 0.1565).

Furthermore, 1.7% (n = 10) of the 573 samples from mammals and 1.5% (n = 3) of the 199 samples from birds tested positive for *Y. pseudotuberculosis*. No significant difference was calculated (*p* = 1.000).

*Y. pseudotuberculosis* was isolated from 1.7% (8 of 459) of the carcasses from the Wilhelma Zoo and 16.7% (3 of 18) of the carcasses from the Opel Zoo. Using the exact Pearson chi-squared test, this difference was statistically significant (*p* = 0.006). In contrast, none of the fecal samples in the Wilhelma Zoo were positive, but 1.4% (2 of 141) of the samples at the Opel Zoo tested positive, representing a statistically not significant difference (*p* = 0.228).

In total, 0.7% (n = 2) of the 295 fecal samples and 2.3% (n = 11) of the 477 carcasses from both zoos were positive. The difference in these results is not statistically significant (*p* = 0.147).

During the sample collection period, 311 small wild mammals and birds living in the wild were found dead at the Wilhelma. Of these, 1.9% (n = 6) tested positive for *Y. pseudotuberculosis*. Among the wild mammals found dead, 66.7% (n = 4) were rats, 16.7% (n = 1) represented a brown hare, and 16.7% (n = 1) represented an Eurasian red squirrel. All small mammals that tested positive were found dead in enclosures where a ‘hotspot species’ lived.

Although more than half of the animals examined were mice, *Y. pseudotuberculosis* could not be detected in any of the carcasses of these animals.

**Figure 1:**
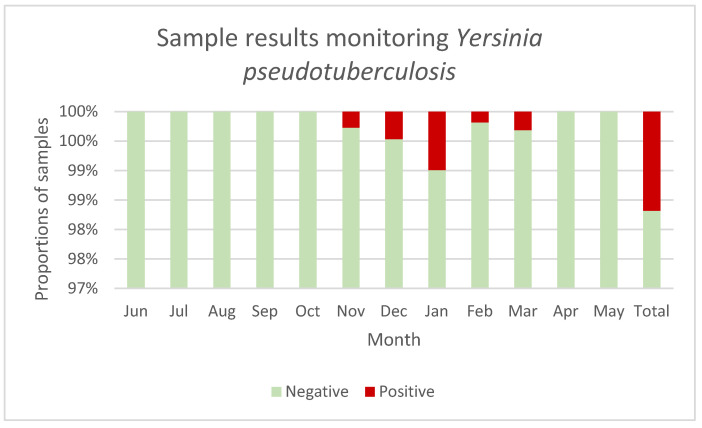
Results of a total of 772 monitoring samples from the Opel Zoo and Wilhelma Zoo taken from June 2023 to May 2024.

Evaluations of all data available for this study regarding the seasonal occurrence revealed that *Y. pseudotuberculosis* infections occurred significantly more frequently from November to March than in the other months of the year (*p* = 0.002) (Figure 1).

### 3.2. Occurrence of Pseudotuberculosis in Mammals from All the 19 Zoos and the Private Keeping Included in This Study

The 117 cases of *Y. pseudotuberculosis* in zoo animals collected for the study came, with few exceptions, from the mammalian orders of Rodents, Primates, and Artiodactyls, especially ruminants. There were also few cases in the mammalian orders of Carnivores, Diprotodontes, Hyracoides, and Macroscelides.

Various orders and species of *Y. pseudotuberculosis* infections were also found in the bird class. Fatal cases occurred mainly in the orders Passeriformes, Piciformes, and Psittaciformes.

#### 3.2.1. Rodents

Exotic rodent species kept in zoological institutions are sensitive to infection with *Y. pseudotuberculosis*. This becomes clear based on 23 pathological reports on rodents that died from acute *Y. pseudotuberculosis* infections in the zoos included in this study (Table 2). The affected rodents belonged to seven different species. During postmortem examinations, high-grade *Y. pseudotuberculosis* infections were detected that affected multiple organs, especially the liver, lungs, kidneys, spleen, intestines, and the associated lymph nodes. These organs showed multifocal, purulent-to-necrotizing gross lesions.

Looking at the seasonal occurrence of cases of *Y. pseudotuberculosis*, it is striking that all but one case (August 2017) occurred during the cold season between October and April.

#### 3.2.2. Primates

**Figure 2:**
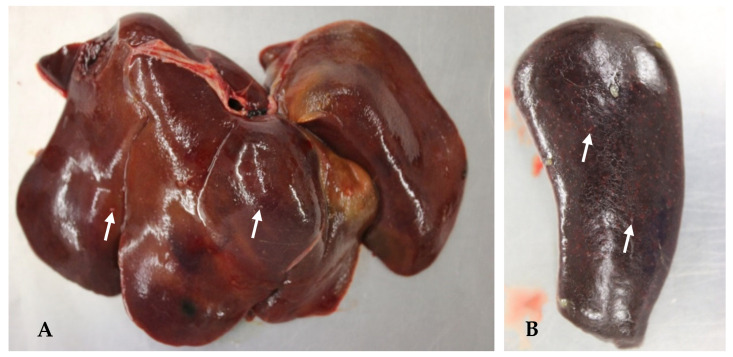
Liver (**A**) and spleen (**B**) of a patas monkey (*Erythrocebus patas*) infected with *Y. pseudotuberculosis* with multifocal miliary lesions (two lesions of each organ are marked with white arrows). *Y. pseudotuberculosis* was isolated from both organs by bacteriological examination. Photos (**A**,**B**): Martin Peters, CVUA Westphalia.

**Figure 3:**
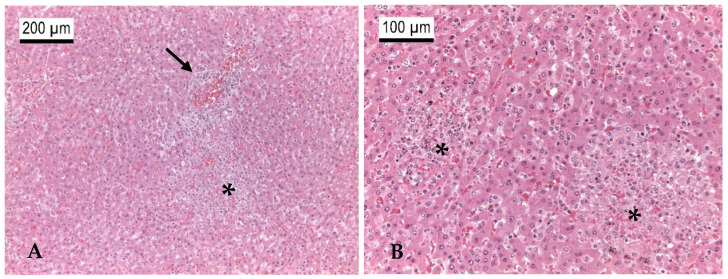
Histo-pathological sections of the liver of a patas monkey (s. Figure 2) with mild periportal lymphocytic infiltrates (black arrow) (**A**) and acute irregular necrotic to pyogranulomatous lesions (black stars) (**A**,**B**) as depicted in the higher magnification (**B**). H&E staining; 100× magnification (**A**), 200× magnification (**B**). Photos (**A**,**B**): Martin Peters, CVUA Westphalia.

**Figure 4:**
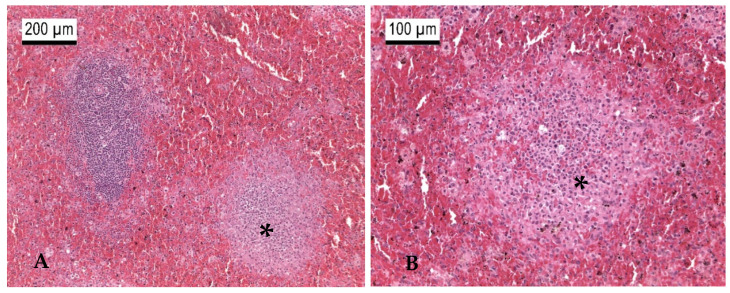
Histo-pathological sections of the spleen of a patas monkey (s. Figure 2). Severe congestion and multifocal acute coagulation necrosis (black star) (**A**) and pyogranulomatous foci depicted in the higher magnification (**B**). H&E staining at 100× magnification (**A**) and 200× magnification (**B**). Photos (**A**,**B**): Martin Peters, CVUA Westphalia.

**Figure 5:**
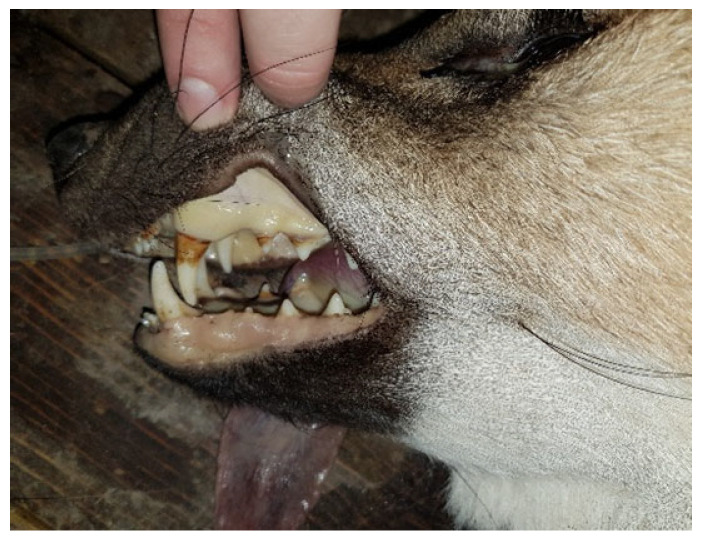
Maned wolf with icteric mucous membranes. Photo: Marco Roller, Zoo Karlsruhe.

Between 2013 and 2023, several German zoos reported mortalities in primates from eleven different species due to infections with *Y. pseudotuberculosis* (Table 3). Black spider monkeys (*Ateles fusciceps rufiventris*), Geoffroy’s spider monkeys (*Ateles geoffroyi*), emperor tamarins (*Saguinus imperator*), spring tamarins (*Callimico goeldii*), cotton-headed tamarins (*Saguinus oedipus*), and representatives of the capuchin-like squirrel monkeys (*Saimiri sciureus*) were particularly frequently affected, as well as bonobos (*Pan paniscus*). The animals often died within a few hours to a few days after the onset of non-specific signs such as apathy or respiratory abnormalities. Therapeutic attempts were usually unsuccessful. During postmortem examinations, multifocal, miliary abscesses were found in the liver, spleen, lungs, intestines, and mesenteric lymph nodes in most cases, which were loaded with high-grade amounts of *Y. pseudotuberculosis* (Table 3). These typical changes were seen macroscopically and histologically in a patas monkey (*Erythrocebus patas*) infected with *Y. pseudotuberculosis* (Table 3, P17; Figure 2, Figure 3, Figure 4 and Figure 5).

There were various therapeutic attempts. In 2017, a 39-year-old female bonobo (Table 3, P7) who was nursing a young animal fell ill at the Wilhelma Zoo. The bonobo’s health status had been slowly deteriorating for a few weeks. Despite eating, she continued to lose weight, occasionally had diarrhea, and became more separated from the group. The reason for this was mainly seen in lactation. As a countermeasure, she was fed high-calorie food and multivitamins. The general health condition continued to deteriorate, and the bonobo showed respiratory signs with cough without sputum, wheezing, apathy, and fever. Therapy with the antimicrobial substance azithromycin (Zithromax dry juice for children, azithromycin 1500 mg/mL, Pfizer Pharma GmbH, Berlin, Germany) and antiphlogistic ibuprofen juice (Ibuflam 40 mg/mL ibuprofen, Zentiva Pharma GmbH, Frankfurt/Main, Germany) was started. The condition worsened, and the treatment was supplemented with cortisone dexamethasone (Dexamethason 4 mg/mL, Bela-Pharm GmbH & Co., KG, Vechta, Germany). On the fourth day after the onset of respiratory signs, the bonobo was found dead in the morning. Pathological examination revealed miliary necroses in the lungs and multifocal colliquation necroses with a margin of extracellular bacterial foci and inflammatory cells in the liver and spleen. *Y. pseudotuberculosis* was grown as a pure culture in high numbers from the lungs, liver, liver abscess, and kidneys. Due to pseudotuberculosis in the mother, a fecal sample from the child and two collective fecal samples from the bonobo group were also tested for *Y. pseudotuberculosis*. The pathogen was isolated from the fecal sample of the young bonobo (Table 3, P8). Conversely, the two collected pooled fecal samples from several animals in the remaining group were negative. Since none of the animals showed any clinical signs, no treatment was carried out. Furthermore, no further losses in the animal group were observed within the following seven years.

In December 2019, a female black-capped squirrel monkey (*Saimiri sciureus*; Table 3, P1) was apathetic and showed respiratory signs at the Wilhelma Zoo. The monkey was treated with ceftiofur (Naxcel, ceftiofur 200 mg/mL, Pfizer Pharma GmbH, Berlin, Germany) and isotonic sodium chloride solution (B. Braun SE, Melsungen, Germany) as liquid substitute. Nevertheless, the monkey died in the following night. During postmortem examination, necrotizing multifocal hepatitis, splenic hyperplasia with miliary-necrotizing inflammation, and an intestine with diphtheroid-necrotizing plaques were found. *Y. pseudotuberculosis* was detected in its liver, spleen, lungs, and intestine.

In February 2020, a male brown spider monkey (*Ateles hybridus*) in the Neuwied Zoo was apathetic and had a fever (39.6 °C) and respiratory signs (Table 3, P10). The spider monkey was taken to a veterinary clinic and received an antimicrobial treatment with amoxicillin/clavulanic acid (Synulox 50 mg [40 mg amoxicillin/10 mg clavulanic acid], Zoetis Germany GmbH, Berlin, Germany) and the non-steroidal anti-inflammatory drug (NSAID) meloxicam (Metacam 0.5 mg/mL, Boehringer Ingelheim Vetmedica GmbH, Rosenheim, Germany). The animal died the next morning, and the pathological examination revealed multifocal necrotizing hepatitis and splenitis, necrotizing myelitis with bacterial foci visible in patho-histology, and diffuse catarrhal enteritis.

Furthermore, there was a third case at the Wilhelma Zoo in December 2022. A female Goeldie’s marmoset (*Callimico goeldii*) was apathic and had a cough (Table 3, P16). Therefore, the monkey was treated with the antimicrobial enrofloxacin (Enro-K 25 mg/mL, bela-pharm GmbH & Co., KG, Vechta, Germany), with the NSAID meloxicam (Inflacam 1.5 mg/mL, Wirtschaftsgenossenschaft deutscher Tierärzte eG (WDT), Garbsen, Germany) and received a supportive treatment with bisolvon powder (Bisolvon 10 mg/g, Boehringer Ingelheim Vetmedica GmbH). The monkey died one day later. In the post-mortem examination, it showed multifocal pyogranulomatous hepatitis, diphtheroid-necrotizing enterocolitis with lymph follicle proliferation, and moderate splenomegaly.

*Y. pseudotuberculosis* was detected in the feces of a Black spider monkey (*Ateles fusciceps rufiventris*) and a bonobo (*Pan paniscus*) whose partners had recently died of acute pseudotuberculosis. The animals, from which the feces samples were taken, showed no clinical signs (Table 3, P6 and P8).

#### 3.2.3. Ruminants

The participating zoos provided data on 23 cases of infections with *Y. pseudotuberculosis* in ruminants (Ru) from 12 different species between 2011 and 2024 (Table 4).

**Table 4:** Information on ruminant species (Ru), year, month, gross pathological findings, and matrices from which *Yersinia pseudotuberculosis* was isolated and the zoo of origin.

Case No.	Species	Year	Month	Pathology	Source of *Y. pseudotuberculosis*	Zoo
Ru1	African dwarf goat *Capra aegagrus hircus*	2015	March	Liver multifocal and spleen with focal acute purulent-necrotizing inflammation, severe acute necrotizing ileitis and colitis	Intestine, mesenteric lymph nodes, spleen, liver, lungs	Kronberg
Ru2	Alpine ibex *Capra ibex*	2024	April	Multifocal necrotizing placentitis	Amniotic sac, stomach, liver, lungs, kidney of the fetus	Nuremberg
Ru3	Alpine ibex *Capra ibex*	2024	April	Abortion	Feces	Nuremberg
Ru4	Bactrian deer *Cervus hanglu bactrianus*	2020	December	Liver multifocal single cell necrosis, low-grade acute multifocal erosive-necrotizing ruminitis with intralesional bacteria, chronic multifocal purulent-necrotizing stomatitis, external ear with severe chronic purulent-necrotizing dermatitis	Liver, spleen, mucous membranes of the mouth	Kronberg
Ru5	Blackbuck *Antilope cervicapra*	2018	April	Purulent-necrotizing inflammation in the lungs, liver, intestines, lymph nodes and in the navel area, bacterial foci in the spleen, hyperemia in the brain	Liver, lungs, spleen, kidney	Kronberg
Ru6	Blackbuck *Antilope cervicapra*	2022	November	Multifocal, acute purulent-necrotizing inflammations with bacterial foci in the liver, spleen, kidneys, intestine, mesenteric lymph nodes, heart, and muscles; parasitosis with coccidia	Intestine	Kronberg
Ru7	Blackbuck *Antilope cervicapra*	2023	February	Multifocal necrotizing hepatitis, lymphadenitis, and catarrhal enteritis	Spleen, kidney, lungs, bone marrow	Kronberg
Ru8	Blackbuck *Antilope cervicapra*	2018	January	Multifocal necrotizing hepatitis and nephritis	Liver, lungs, kidneys, brain, intestine	Karlsruhe
Ru9	Blackbuck *Antilope cervicapra*	2020	March	Acute purulent-necrotizing hepatitis and pneumonia	Liver, lungs, leptomeninx, myocardium	Karlsruhe
Ru10	Blackbuck *Antilope cervicapra*	2021	February	Multifocal purulent-necrotizing pneumonia and hepatitis; high-grade embolic-purulent focal nephritis	Liver, lungs, kidney	Karlsruhe
Ru11	Blackbuck *Antilope cervicapra*	2023	January	Acute purulent-necrotizing hepatitis, pneumonia and enteritis	Liver, lungs, intestine	Karlsruhe
Ru12	Blackbuck *Antilope cervicapra*	2023	February	Diptheroid-necrotizing enteritis, parasitosis with gastrointestinal strongylids	Lungs, intestine	Karlsruhe
Ru13	Fallow deer *Dama dama*	2021	February	Acute diffuse fibrinous-necrotizing enterocolitis, acute multifocal purulent-necrotizing lymphadenitis of the mesenteric lymph nodes	Intestine, mesenteric lymph nodes, feces	Zurich
Ru14	Impala *Aepyceros melampus*	2018	January	Brain, lungs, lymph nodes, heart, spleen, liver, kidney, pancreas, intestine with moderate to severe multifocal embolic and purulent partly necrotizing inflammation	Liver, spleen, kidney, lungs, intestine, brain	Kronberg
Ru15	Impala *Aepyceros melampus*	2022	November	Purulent-necrotizing hepatitis and enteritis, lymph node hyperplasia	Liver, spleen, kidney, lungs, intestine, lymph nodes	Kronberg
Ru16	Impala *Aepyceros melampus*	2023	December	Necrotizing enteritis and lymphadenitis, darkening of the liver parenchyma	Liver, spleen, kidney, lungs, mesenteric lymph nodes, bone marrow, abdominal cavity	Kronberg
Ru17	Impala *Aepyceros melampus*	2024	January	Purulent-abscessed lymphadenitis, acute catarrhal enteritis, pneumonia, white foci in the kidney	Intestine	Kronberg
Ru18	Impala *Aepyceros melampus*	2020	February	Acute catarrhal-purulent inflammation with necrosis in the liver, lungs, spleen, intestines; mesenteric lymph nodes	Intestine	Kronberg
Ru19	Markhor *Capra falconeri*	2015	n.a. ^1^	Catarrhal enteritis, parasitosis with coccidia	Intestine	Berlin
Ru20	Mesopotamian fallow deer *Dama mesopotamica*	2020	December	Acute purulent inflammation with necrosis in the liver, lungs, kidneys, spleen, intestines, lymph nodes	Liver, lungs, spleen, kidney	Kronberg
Ru21	Mhorr’s gazelle *Nanger dama mhorr*	2011	n.a. ^1^	Purulent-necrotizing mastitis, splenic hyperplasia and splenomegaly, liver with multiple white foci, purulent endometritis	Liver, spleen, udder, uterus	Berlin
Ru22	Pudu *Pudu puda*	2019	April	Generalized swelling of the lymph nodes, multifocal purulent-necrotizing hepatitis	Liver, lungs, spleen	Wuppertal
Ru23	Pudu *Pudu puda*	2019	January	Enterocolitis	Intestine	Wuppertal
Ru24	Reindeer *Rangifer tarandus*	2021	September	Multifocal acute purulent hepatitis and pneumonia, mesenteric lymph nodes with bacterial foci, pyloric stenosis of the abomasum, hemosiderosis of the spleen	Liver, lungs, spleen, kidney	Wuppertal
Ru25	White-lipped deer *Cervus albirostris*	2024	April	Stillbirth still attached to placenta, advanced stage of autolysis	Liver of the fetus	Berlin

Berlin: Tierpark Berlin; Karlsruhe: Karlsruhe Zoo; Kronberg: Opel Zoo Kronberg; Wuppertal: Wuppertal Zoo; Zurich: Institute for Veterinary Pathology (IVP), University of Zurich; ^1^ n.a.: not available.

The majority of ruminants were in poor nutritional condition, and some were infested with endoparasites like coccidia (Table 4, Ru6 and Ru19) and gastrointestinal strongylids (Ru11). The animals, which came from five different zoological facilities, almost all suddenly died in the winter months from November to April, except for one animal (Ru24). Some animals had been diagnosed with a second cause of infection, such as omphalophlebitis (Ru5) or panophthalmia (Ru10). A blackbuck showed signs of illness due to a *Listeria monocytogenes* infection (Ru6). A bactrian deer suffered from a stomatitis and ear infection as well as from diarrhea (Ru4). Consequently, postmortem, *Y. pseudotuberculosis* was isolated in large numbers in multiple organs from these animals.

At the Nuremberg Zoo, three abortions occurred in the herd of Alpine ibex (*Capra ibex*) between the end of March and the beginning of April 2024. In one case of an abortion (Table 4, Ru2), *Y. pseudotuberculosis* was detected by bacterial culture with strong growth in the lungs, liver, and membranes of the fetus. The fetuses of the other two cases of abortions tested negative. Following the *Yersinia*-positive abortion, a collective fecal sample was taken from the ibex herd. The fecal sample (Table 4, Ru3) was also positive for *Y. pseudotuberculosis*. In the following period, no losses were observed among the lambs and adults in the ibex herd.

In April 2024, an approximately five-year-old female white-lipped deer (*Cervus albirostris*) at the Tierpark Berlin (Table 4, Ru25) showed hemorrhagic vaginal discharge and increased abdominal clenching. Otherwise, the animal showed no clinical signs nor signs of birth. Due to the clinical signs, the animal was immobilized and diagnosed to be in the birthing phase. Therefore, a cesarean section was performed and a dead, immature fetus, which was still attached to the placenta, was delivered. Bacteriological examination of the stillbirth revealed a high-grade growth of *Y. pseudotuberculosis* in the bacterial culture of the liver.

Another case of *Y. pseudotuberculosis* occurred in a female Mhorr’s gazelle (*Nanger dama mhorr*) in connection with a birth in 2011 (Table 4, Ru21). After giving birth to a weak fawn, the gazelle showed lochial discharge and therefore was treated with amoxicillin (Vetrimoxin LA 150 mg/mL, Ceva animal health GmbH, Dusseldorf, Germany) twice at intervals of two days. Despite the two treatments, the animal showed a moderately reduced general condition, and therefore, the therapy was continued with marbofloxacin (Marbox 100 mg/mL, Ceva animal health GmbH) per day. However, a week after the last application of antimicrobials, the animal’s general condition deteriorated significantly. Thus, the treatment with marbofloxacin was repeated, but the animal had to be euthanized on the second day. Pathological examination revealed multifocal acute purulent-necrotizing inflammation in the liver, spleen, udder, and uterus of the Mhorr’s gazelle. *Y. pseudotuberculosis* was detected in all organs mentioned.

#### 3.2.4. Other Mammal Species

##### Carnivora

**Figure 6:**
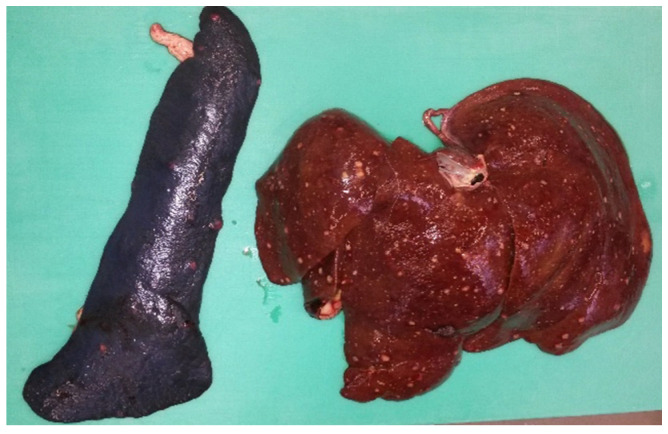
Spleen and liver of the maned wolf with multiple abscesses. Photo: CVUA Stuttgart.

At the Wilhelma Zoo, a female maned wolf (*Chrysocyon brachyurus*) fell ill with a *Y. pseudotuberculosis* infection in December 2017. Previously, the animal had shown a reduced food intake for two days, had been increasingly apathic, and had diarrhea since the first day of illness. When examined under anesthesia, the mucous membranes were severely jaundiced, and the animal was severely dehydrated (Figure 5). In the abdominal sonography, liver cysts were suspected, and in the blood biochemestry, the kidney values for creatinine (CREA: value 150 µmol/L [normal range 27–124 µmol/L]) and blood urea nitrogen (BUN: value 29.2 µmol/L [normal range 2.5–8.9 µmol/L]) were highly elevated. This also applied for the liver values for alanin-amino-transferase (ALT: value 7951 nkat/L [normal range 167–1967 nkat/L]) and total bilirubin (TBIL: value 58.2 µmol/L [normal range 1.7–10.2 µmol/L]) accompanied by an anemia (erythrocyte count RBC: value 3.4 T/L [normal range 5.5–8.5 T/L]; the hematocrit HCT value was 0.26 L/L [normal range 0.37–0.55 L/L]). Due to the poor prognosis, the maned wolf was euthanized and subjected to postmortem examination. The maned wolf was in poor nutritional condition. The inspection of the inner organs revealed a hepatitis and splenitis with disseminated capsular neutrophilic microabscesses and coagulation necroses detected by patho-histology (Figure 6). In addition, ulcerative inflammations in the stomach and intestinal mucosa with superficial epithelial loss were found. *Y. pseudotuberculosis* was detected culturally with dense growth from the microabscesses.

##### Diprotodontia

**Figure 7:**
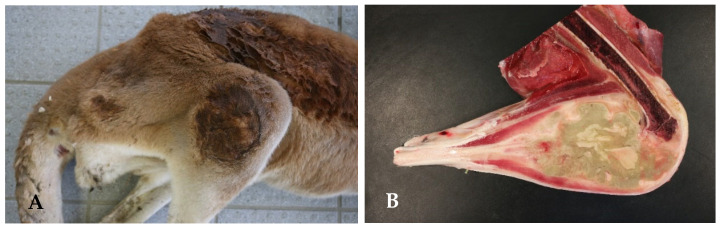
Severely swollen knee joint with skin ulcer (**A**) and its respective cut section (**B**) of a red kangaroo (*Osphranter rufus*) with purulent gonitis caused by *Y. pseudotuberculosis*. Photos: Martin Peters, CVUA Westphalia.

At the Dortmund Zoo, in the period from 2013 to 2015, four red kangaroos (*Osphranter rufus*) developed motion disorders with hind limb lameness and weight loss. All four kangaroos were euthanized due to the severity of the disease. Postmortem examination revealed unilateral severe subacute to chronic purulent destructive coxarthritis in three animals and unilateral severe purulent gonitis with osteomyelitis in the fourth animal (Figure 7). The youngest of the four kangaroos was euthanized at the age of eight months with proliferative synovitis of the left hip joint and an abscess at the edge of the pouch. The oldest of the kangaroos was five years old and, in addition to the joint alterations, had formed an intestinal lymph node abscess. The altered joints of the four kangaroos, the pouch abscess from the youngest, the intestine, and the intestinal lymph node of the oldest animal were examined bacteriologically. *Y. pseudotuberculosis* could be cultivated from all samples.

During the disease outbreak, two employees at the Dortmund Zoo developed a reactive arthritis. One of them had been temporarily in charge of the kangaroos. Both keepers were positive for anti-*Yersinia* IgG using a strip immunoassay.

At the Berlin Zoo, fatal cases of *Y. pseudotuberculosis* infection occurred in two western gray kangaroos (*Macropus fuliginosus*) in 2019 and 2021, respectively. In the first case in 2019, a female adult kangaroo was found in the morning with trembling, followed by an acute collapse and recumbency in the afternoon. The kangaroo was treated with an antimicrobial (Procain-Penicillin Susp. 300 mg/mL, Dechra Veterinary Products, Aulendorf, Germany) intramuscularly and cortisone (Prednisolone acetate injection suspension 10 mg/mL, CP-Pharma Handelsges. GmbH, Burgdorf, Germany). However, the animal was found dead the next day. The postmortem examination revealed a poor nutritional condition, and inspection of the abdominal cavity exhibited highly purulent, abscessing mesenteric and pyloric lymphadenitis and moderate serosanguinous ascites with lots of fibrin flakes. The bacteriological examination yielded *Y. pseudotuberculosis* in a dense culture.

In the second case in 2021, a female, juvenile western gray kangaroo died after an attack from an adult buck despite lacking external injuries. The postmortem examination revealed numerous pathological changes such as poor nutritional status, splenitis, and inflamed, enlarged lymph nodes in the intestine, groin, and esophagus. The liver displayed a fine bumpy surface interspersed with fine white-gray dots. Further changes included an abdominal cavity effusion and an erosive to ulcerative gastritis. High loads of *Y. pseudotuberculosis* and, as a secondary finding, *Salmonella enterica* (serogroup B) could be detected in bacterial culture obtained from the mesenteric lymph nodes, spleen, liver, stomach, ileum, cecum, and effusion.

In August 2024, at the Erfurt Zoo, a female adult western gray kangaroo showed a lack of appetite and poor general health. On the second day of illness, the animal was anaesthetized and examined in general with an additional sonographic examination. In the kidneys, stones were found and, as a secondary finding, an enlargement of the abdomen. The animal was euthanized, and a postmortem examination was carried out. A purulent abscess of the intestinal lymph node measuring 15 × 8 × 8 cm proved to be the cause of the enlarged abdomen, from which *Y. pseudotuberculosis* could be isolated. The animal also suffered from nephropathy with medullary and papillary necrosis with high-grade renal pelvic stone formation on both sides and, additionally, mitral valve endocardiosis.

##### Hyracoidea

In April 2015, a female bush hyrax (*Heterohyrax brucei*) suddenly died at the Opel Zoo. Postmortem examination of the cachectic animal revealed severe purulent-necrotizing hepatitis, rhinitis, and lymphadenitis of the mesenteric lymph nodes. In the latter as well as in the bone marrow and spleen, large numbers of microabscesses were found in all organs, from which *Y. pseudotuberculosis* was isolated in high numbers.

##### Macroscelidea

In January 2020 as well as in November 2022, a female adult short-eared elephant shrew (*Macroscelides proboscideus*) died suddenly in the Wilhelma Zoo without any previous abnormalities. The animal, which died in 2020, was in a good nutritional condition. Pathological examination revealed massive bacterial emboli in the heart with high-grade consecutive multifocal purulent-embolic myocarditis. The animal also suffered from a mild acute diffuse panlobular purulent hepatitis. The kidneys showed severe acute necrotizing tubulonephritis. *Y. pseudotuberculosis* could be obtained in culture from the liver, spleen, and kidneys.

In 2022, another short-eared elephant shrew died at the Wilhelma Zoo. The animal had a moderate nutritional condition. The histo-pathological examination revealed moderate fatty degeneration of the hepatocytes with miliary necroses and bacterial foci. In the small intestine, hemorrhagic enteritis with low-grade mononuclear infiltration of the mucosa was observed. *Y. pseudotuberculosis* could be cultured with high-grade growth from the liver, lungs, kidneys, and small intestine.

In November 2017, a male and a female short-eared elephant shrew died within five days without prior clinical signs at the Wuppertal Zoo. During postmortem and histological examinations of the organs, both animals showed multifocal miliary necroses with bacterial foci in the liver. High bacterial loads of *Y. pseudotuberculosis* were detected by culture from the liver, spleen, kidneys, and lungs.

### 3.3. Occurrence of Pseudotuberculosis in Birds from All the 19 Zoos Included in This Study

Birds of ten different orders and 25 species proved to be infected with *Y. pseudotuberculosis* (Table 5). In all cases, the zoos participating in this study reported sudden cases of death among them devoid of prior clinical signs. During the pathological examinations, the main findings in these animals were high-grade multifocal necroses in the liver and lungs but also in the spleens and kidneys, which were loaded with foci of rod-shaped bacteria and granulomas consisting of heterophils, lymphocytes, histiocytes, and multinucleated giant cells visible in histo-pathology. In addition, some of the affected birds suffered from catarrhal enteritis with lymphadenitis or granulomatous pneumonia. High loads of *Y. pseudotuberculosis* could be detected by culture in all affected organs (Table 5).

In February 2023, two toco toucans (*Ramphastos toco*) (Table 5, B37 and B38), which had hatched in Walsrode in spring 2022, died within a few days. The birds had been vaccinated twice, four weeks apart, with a flock-specific vaccine against *Y. pseudotuberculosis* and did not have access to the outdoor enclosure since October 2022. The indoor aviary was safe from rodents. One toucan died suddenly (Table 5, B37), and the other toucan (Table 5, B38) showed wheezing in the morning; therefore, an antimicrobial treatment with enrofloxacin (Enrofloxacin 2.5% WDT injection solution) intramuscularly administered was initiated. However, the bird died a few minutes later. During the pathological examination, highly multifocal necroses with patches of rod-shaped bacteria were found in the liver, lungs, and spleen of both toucans. High levels of *Y. pseudotuberculosis* were isolated from the liver, lungs, blood, and body cavity.

In November 2023, an eastern rosella (*Platycercus eximius*) (Table 5, B12) and a red-rumped parrot (*Psephotus haematonotus*) (Table 5, B31) died suddenly at the Wilhelma Zoo within five days from an infection with *Y. pseudotuberculosis*. The birds lived in the same outdoor free-flight aviary as the red-rumped parrot that had been considered a ‘hotspot species’, since it died suddenly from an infection with *Y. pseudotuberculosis* in January 2023 (Table 5, B30). Due to repeated cases of pseudotuberculosis, a collective fecal sample from the aviary was examined for *Y. pseudotuberculosis* every month starting from June 2023. However, all samples examined over the entire study period of one year were yielded negative results, including those taken immediately after the first animal from the aviary had died in November 2023. During pathological examinations, all three birds that died in 2023 showed moderate to poor nutritional conditions, miliary necrotizing granulomatous hepatitis, and splenitis with extracellular bacterial foci. Two animals suffered from pyogranulomas in the lungs, catarrhal enteritis, and granulomatous-necrotizing encephalitis. Already in November 2022, a spotted dove (*Spilopelia chinensis*) (Table 5, B32) and in January 2021 an adult common redshank (*Tringa totanus*) (Table 5, B11) from the same aviary died suddenly from *Y. pseudotuberculosis* infections. *Y. pseudotuberculosis* isolates were obtained from the liver, heart, and lungs of these birds. In addition, a brown rat (*Rattus norvegicus*) (Table 2, R1), a brown hare (*Lepus europaeus*), and an Eurasian red squirrel (*Sciurus vulgaris*) (Table 2, R11) were found dead in or very close to the outdoor aviary area during the study period. Organ samples of the animals tested positive for *Y. pseudotuberculosis*.

The isolates obtained from the zoo animals were cryopreserved, and a flock-specific vaccine was produced by Ceva BESTVAC (Dessau-Roßlau, Germany) on demand. This vaccine was administered at the Wilhelma Zoo starting in January 2024. All birds in the outdoor free-flight aviary were vaccinated in February and four weeks later in March. Birds that were newly integrated into the existing population were given a basic immunization during quarantine.

In January 2024, a female lesser flamingo (*Phoeniconaias minor*) died suddenly at the Opel Zoo (Table 5, B21). The bird suffered from cachexia and ascites, and granuloma-like proliferations were visible during postmortem examination. In addition, beige yellow pinhead-sized foci were found on the serous membranes. In the liver parenchyma, several beige yellow partly raised foci were visible. *Y. pseudotuberculosis* was detected by bacteriological examination in high numbers from in the kidneys, lungs, heart, ovary, and to a low degree from the liver.

### 3.4. Antimicrobial Susceptibility

The total of 270 *Y. pseudotuberculosis* isolates, comprising 132 from zoos and 138 from wild animals, were investigated for their antimicrobial resistance profiles. All isolates were not highly sensitive to erythromycin. In 92 antimicrobial susceptibility tests, no inhibition zone was observed. In 153 antibiograms, the inhibition zone was less than or equal to 10 mm in diameter. Only in 25 antibiograms was there an inhibition zone larger than 10 mm in diameter. However, the *Y. pseudotuberculosis* isolates were more sensitive to florfenicol than to erythromycin. Thus, florfenicol showed no inhibition zone in only two antibiograms, an inhibition zone of less than or equal to 10 mm in one antibiogram, and an inhibition zone with a diameter of greater than or equal to 25 mm in the remaining 267 antibiograms. None of the inhibition zones for florfenicol were in the range between 11 mm and 25 mm diameter. The results of the tests on other antimicrobial agents with available reference values from the CLSI version VET01-SEd7E guidelines for agar diffusion test are summarized in Table 6.

## 4. Discussion

*Y. pseudotuberculosis* infections have a significant impact on animal health and, due to their zoonotic nature, on humans who come into close contact with animals or environmental contamination. Pseudotuberculosis is therefore an infectious disease that needs to be thoroughly investigated within the One Health approach, which includes humans, animals, and the environment. Since fatal infections with *Y. pseudotuberculosis* occur repeatedly in numerous zoos, the aim of the present study was to investigate the prevalence of and the impact of *Y. pseudotuberculosis* infections on such special epidemiological units.

As part of this study, numerous case reports of *Y. pseudotuberculosis* infections were collected and analyzed based on accessible literature. This overview was supplemented by cases which were personally communicated from zoo veterinarians from other zoos. Many cases were available from zoo animals of the orders of rodents (Table 2), primates (Table 3), and ruminants (Table 4), as well as from birds of the order Piciformes (Table 5).

### 4.1. Y. pseudotuberculosis Infections in Zoo Animals

The regular occurrence of *Y. pseudotuberculosis* in rodents like capybaras, maras, and guinea pigs is to be expected, since cases of Y. pseudotuberculosis infections in capybaras kept in zoos have been described previously [28,29]. During this study, cases also occurred in rodents in several German zoos, in particular in Patagonian maras (*Dolichotis patagonum*) in the Wuppertal Zoo (Table 2, R9–R10, R15–R18).

Cases of *Y. pseudotuberculosis* have also occurred in various primate species in several zoos, for example, in a bonobo at the Wilhelma Zoo (Table 3, P7 and P8) and mainly in non-human primates in other zoos in recent years. The importance of *Y. pseudotuberculosis* in primates is corroborated by numerous published case reports on cases in meerkats [30,31], a marmoset [32], cynomolgus monkeys [33,34,35], baboons and patas monkeys [35,36], spider monkeys [36], squirrel monkeys [31,37], and rhesus monkeys [38].

In recent years, there have also been cases of *Y. pseudotuberculosis* in different ruminant species at the Opel Zoo like in a fallow deer (*Dama mesopotamica*) and in impalas (*Aepyceros melampus*) (Table 4, Ru1, Ru4–Ru6, Ru12, Ru14–Ru18, Ru20). But there are not only wild ruminants that are susceptible to the pathogen. Domestic ruminants such as goats and sheep, which are very popular in petting zoos, are susceptible and can be sources of infections. This was proven by a case of a *Y. pseudotuberculosis* infection in an African dwarf goat at the Opel Zoo in 2015 (Table 4, Ru1). As part of various studies in Australia, *Y. pseudotuberculosis* was detected in sheep feces, and some of the animals suffered from diarrhea [23,24,25,26]. Enteritis caused by *Y. pseudotuberculosis* has also been reported for goats [39]. Moreover, goats were reported in a retrospective study to be the species that was mostly affected in California (USA) by this pathogen [40]. It should be kept in mind that many visitors, especially children, pet sheep and goats and thus potentially come into close contact with feces contaminated with *Y. pseudotuberculosis* and other numerous zoonotic pathogens [41,42,43].

Beyond enteral infections, cases of abortion and stillbirth in small ruminates caused by *Y. pseudotuberculosis* infections are documented [44,45,46]. Like the cases in the Alpine ibex herd at the Nuremberg Zoo, cumulated cases of abortion and stillbirth have been reported in small ruminants [47,48,49]. Thus, massive excretion of *Y. pseudotuberculosis* via lochia and placenta causing heavy environmental contamination should be considered in hygiene concepts [44].

Due to the wide host spectrum of *Y. pseudotuberculosis*, not only mammals but also various bird species are affected, thereby causing animal losses. During the monitoring period, *Y. pseudotuberculosis* infections were detected in a red-rumped parrot (*Psephotus haematonotus*) and an eastern rosella (*Platycercus eximius*) from the Wilhelma Zoo as well as in a lesser flamingo (*Phoeniconaias minor*) from the Opel Zoo. Some of these birds are still ‘hotspot species’. Cases of pseudotuberculosis in birds have been reported for numerous species such as toucans [2,40], crows [50], Amazon parrots (*Amazona aestiva* and *Amazona oratrix*) [51], pigeons (*Nesoenas mayeri*) [52], doves (*Streptopelia decaocto*) [53], and a bustard (*Otis tarda*) [54].

However, during our study, confirmed lethal infections also occurred in species that have not yet been recognized to be highly susceptible to *Y. pseudotuberculosis* infections. These include a maned wolf (*Chrysocyon brachyurus;* Wilhelma), short-eared elephant shrews (*Macroscelides proboscideus*; Wilhelma Zoo and Wuppertal Zoo), red kangaroos (*Osphranter rufus*; Dortmund Zoo) and lesser flamingo (*Phoeniconaias minor;* Opel Zoo; Table 5, B21). Similarly, several individual cases of pseudotuberculosis have been described in species in which infections were hardly expected, i.e., in two adult male lions (*Panthera leo*) in a zoo in North Carolina and infections in a colony of Egyptian fruit bats (*Rousettus aegyptiacus*) in Egypt [55,56]. This means that in epidemiological units such as zoos, the detection of *Y. pseudotuberculosis* in key species represents the tip of the iceberg, and thus, many other animals could be at risk.

The high lethality rate in all infected animal species associated with sudden deaths seldom preceded by unspecific clinical signs such as apathy or anorexia and consecutive treatments appeared to have had no prospects of success. Thus, *Y. pseudotuberculosis* infections were only diagnosed after death at the Wilhelma Zoo and the Opel Zoo in accordance with previous reports from other zoos [46,50,51,52,57]. In the London Zoo and the Whipsnade Zoo, 24 cases of *Y. pseudotuberculosis* infections occurred between 2001 and 2019 in primates, artiodactyls, and birds. Common clinical signs again included lethargy or death without prior notice. Poor general condition was common in mammals but often went undetected until postmortem examinations. Bacterial cultures were often obtained from the liver or spleen of primates and birds or from enlarged mesenteric lymph nodes in artiodactyls after postmortem examinations [21]. In addition to changes in internal organs such as the lungs, spleen, and liver, inflammations in the intestine and the mesenteric lymph nodes are striking findings in many perished animals. This indicates a rapid spread of pathogenic *Y. pseudotuberculosis* bacteria from the intestine via the lymph nodes due to a tropism to the lymphatic system and subsequent colonization of the internal organs [58,59].

In addition to the examinations of deceased zoo animals, a total of 168 fecal samples from ‘hotspot species’ were tested for *Y. pseudotuberculosis* on a monthly base for one year. Although these samples came from areas at the Wilhelma Zoo and the Opel Zoo where zoo animals had previously died of *Y. pseudotuberculosis* infection, only one sample from the Opel Zoo was found positive from an impala. A second positive fecal sample was taken from a healthy blackbuck as part of a transport requirement. Fecal samples from the Alpine ibex herd at the Nuremberg Zoo, in which *Y. pseudotuberculosis* was involved in several cases of abortions, tested positive. However, the majority of fecal samples must be regarded as negative like in the pooled fecal sample taken from the free-flight aviary at the Wilhelma Zoo, albeit a singing parakeet had died there on the same day. Another case was recorded at the Wilhelma Zoo in connection with *Y. pseudotuberculosis* infection in a bonobo in 2017. *Y. pseudotuberculosis* was detected in the feces of the offspring whose mother bonobo had died of pseudotuberculosis but not during control samplings within the same group. In total, only 0.7% of the fecal samples tested positive for *Y. pseudotuberculosis* in our study at the Wilhelma Zoo and the Opel Zoo. However, another study achieved higher values for the detection of *Y. pseudotuberculosis* during an outbreak of diarrhea in weaned Merino sheep in Australia. Out of 1,020 fecal samples, *Y. pseudotuberculosis* was the most frequently isolated pathogen (18.5%), followed by virulent *Y. enterocolitica* (13.6%) [60]. Nevertheless, fecal samples do not reliably reveal the shedding of *Yersinia* species [61], and it should be noted that the shedding of the pathogen depends on various factors and is not always associated with obvious clinical signs [46,60,62]. To this end, the spread of this pathogen to the environment through fecal shedding by systemically infected animals should be considered. Outbreaks can result in significant financial losses and have the potential to undermine conservation efforts by leading to increased mortality of threatened and endangered species [46]. Hence, all animals that die in zoos should be subjected to pathological and bacteriological examinations for monitoring purposes.

### 4.2. Y. pseudotuberculosis in Free-Living Animals

As part of a monitoring plan, 312 wild small mammals and birds found dead in and around the Wilhelma were considered as potential carriers and examined for *Y. pseudotuberculosis*. Six small mammals (4 out of 55 rats, one squirrel, and one hare) tested positive, corresponding to a prevalence of 2%. No specific *Y. pseudotuberculosis* proof was achieved in the 156 mice examined. Rats were caught as part of the rodent control program in the zoo from three enclosures where a ‘hotspot species’ lived, but they did not show any pathological organ changes postmortem. During a 2012 outbreak at the national zoo in Israel, which killed 15 zoo animals, no *Yersinia* bacteria were isolated from rodent samples [20]. Similarly, only 2 out of 1,840 free-ranging Finnish mice tested positive over a period of seven years [63], and in southeastern Poland, just 1 out of 214 small wild rodents carried the pathogen [6,7,41]. Nevertheless, rodents are considered asymptomatic vectors and important in the spread of the pathogen. Therefore, rodent control is essential.

However, it should be noted that *Y. pseudotuberculosis* is present in soil and can sometimes be detected in fresh, agriculturally produced plants, and root vegetables.

Thus, *Y. pseudotuberculosis* can infect both humans and animals through the consumption of contaminated food and water or contact with contaminated soil [64,65,66].

### 4.3. Preventive Measures

Zoonoses continue to pose a threat to human and animal health, and pathogen transmission from animals to humans is a key issue of the One Health approach [67,68]. For this very reason, research in this area is relevant, especially for pathogens that have received less attention and have the potential to emerge, particularly in those with already known as well as emerging histories [69].

Zoos represent unique artificial habitats, where a wide variety of animal species from disparate ecosystems live in close quarters, thereby increasing *Y. pseudotuberculosis* dissemination risks among species, including humans. Shedding of the pathogen via feces, particularly from asymptomatic animals, contaminates the environment and facilitates pathogen spread through the fecal–oral route [23]. The zoonotic potential of *Y. pseudotuberculosis* poses health risks to zookeepers and visitors, as illustrated in the Dortmund Zoo, where kangaroo keepers developed reactive arthritis and tested seropositive (L. Riede, personal communication). Detailed investigations and biosafety measures are essential to better protect humans, animals, and the environment.

The most effective measures to prevent infections with *Y. pseudotuberculosis* include regular hand hygiene. The COVID-19 pandemic has left a lasting impression on hand hygiene behavior [70]. Since then, practicing better hand hygiene through more frequent handwashing and sanitizing, especially in public, has been achieved and maintained by many. Therefore, the most effective protective measures include reminding the importance of hand hygiene and providing easy access to handwashing facilities [42,43,71]. At the Wilhelma Zoo, hand wash basins and disinfectant dispensers have been installed for visitors in front of the petting zoo enclosure and in the sanitary facilities.

The National Association of State Public Health Veterinarians (NASPHV, Inc.) and the Centers for Disease Control and Prevention (CDC) have jointly published comprehensive recommendations to reduce zoonotic disease risks associated with animals in public settings. The management of contact between the public and animals is also addressed regarding the design of facilities and animal housings, cleaning procedures, and during veterinary care and animal husbandry [41,72]. Regular hygiene training with a range of topics on zoonotic pathogens is important to ensure occupational safety and to protect the health of the staff.

Vaccination is another option to prevent spreading and is considered the most effective way to prevent and control zoonosis sustainably [73]. However, only a vaccine approved for cervids in New Zealand (Yersiniavax^®^, inactivated *Y. pseudotuberculosis* serotypes I, II, III, MSD Animal Health New Zealand) and a killed whole-cell vaccine used mainly in European zoos (Pseudovac^®^, Department of Veterinary Pathology, Utrecht University, The Netherlands) are currently commercially available. Thus, an autogenous inactivated vaccine has to be used for other purposes. The production and administration of autogenous vaccines must be based on isolates obtained from animals belonging to the same epidemiological entity [74]. These vaccines require the cultivation of current and relevant isolates, as zoo isolates often differ significantly [61]. Furthermore, this kind of vaccine lacks any proof of efficiency and innocuousness and should therefore be used with caution. At the Wilhelma Zoo, an autologous vaccine obtained from an avian *Y. pseudotuberculosis* isolate was produced on demand. Following the depicted losses, this vaccine was used to immunize birds from the outdoor free-flight aviary twice in spring 2024 at intervals of three to five weeks and so far did not have any side effects. An autologous vaccine was also produced at the Opel Zoo and used to vaccinate the impalas and lesser flamingos.

Overall, the use of vaccines against pseudotuberculosis has been controversially discussed. Bakker et al. (2007) thus concluded that vaccination was essential for the control of *Yersinia* spp. outbreaks in monkeys (marmosets and tamas) [32]. However, a study on Merino sheep demonstrated limited vaccine impact on seroconversion or fecal shedding of the pathogen [75]. Similarly, Quintard et al. (2010) found the inactivated vaccine Pseudovac^®^ ineffective in guinea pigs, whereas oral vaccination with attenuated *Y. pseudotuberculosis* strains proved superior [76].

Encouraging data from immunizations based on orally administered attenuated live vaccines such as recombinant YopE vaccine [77], pTTSS mutants, recombinant YopE vaccines [58], and genetically engineered *Lactococcus lactis* used as a vaccine vector that provides the *Y. pseudotuberculosis* immunomodulatory LcrV protein [78,79,80,81] have been presented. These findings emphasize the need for tailored vaccination strategies, especially prior to an increased risk of infection in winter [32,58,60,75].

### 4.4. Antimicrobials

Antimicrobial therapies have proven to only be promising in the very early stages of infection due to the predominantly fulminant course of *Y. pseudotuberculosis* infections in zoo animals and therefore are of minor importance. Nevertheless, antimicrobials remain the treatment of choice in humans, guided by susceptibility testing. The *Y. pseudotuberculosis* isolates obtained in this study showed low levels of antimicrobial resistance in vitro. The isolates were sensitive to most of the tested antimicrobial agents such as amoxicillin/clavulanate, ciprofloxacin, gentamicin, norfloxacin, streptomycin, and trimethoprim/sulfamethoxazole. Of the antimicrobial agents tested on the 270 isolates in our study, resistance was frequently observed in the case of erythromycin, tetracycline, and occasionally to amoxicillin/clavulanate as well as ampicillin. This is a worrying trend, as ampicillin, amoxicillin/clavulanate, and tetracyclines are commonly used agents for a wide range of infections [82]. Alarmingly, 2.2% of the 270 isolates demonstrated resistance to meropenem, a last-resort treatment for multidrug-resistant infections [83]. Similar to the isolates included in this study, isolates obtained from an outbreak of pseudotuberculosis in a zoo in Israel and the London Zoo and Whipsnade Zoo were resistant to tilmicosin and clindamycin, respectively [20,21].

A study on the antimicrobial susceptibility of *Y. pseudotuberculosis* isolated from pig tonsils showed that all isolates were sensitive to all antimicrobials included, except ampicillin, amoxicillin/clavulanate, and tetracyclines, to which resistance occurred in our study [79]. This was also true for *Y. pseudotuberculosis* strains from different human (feces) and non-human (pig, wild boar, monkey, chinchilla, mara, capybara, and lettuce) sources that proved sensitive to all antimicrobial agents of various groups tested [84]. However, in a previous study on an outbreak of diarrhea (winter scours) in Australian merino sheep, 87% of the *Y. pseudotuberculosis* isolates revealed resistance to sulfafurazole [60].

Enrofloxacin is usually an effective antimicrobial for birds. However, the use of enrofloxacin against *Y. pseudotuberculosis* in canaries has been reported with varying degrees of success [85]. On the other hand, two studies performed in 1991 and 2021 reported on the administration of fluoroquinolones as the antimicrobial agent of choice to treat animals suspected to be infected with *Y. pseudotuberculosis* [61,86]. However, fluoroquinolones are last-resort antimicrobial agents that must not be used as first-line treatments but only if no alternatives are available [87]. Furthermore, it should be considered that antimicrobial susceptibility testing should be carried out in order to prevent the development of antimicrobial resistance [86], even though discrepancies between in vitro and in vivo effectiveness of antimicrobial agents occur [61,86]. Overall, the prudent use of antimicrobial agents should be promoted worldwide in the spirit of the One Health approach [82,88,89].

### 4.5. Seasonal Occurrence of Y. pseudotuberculosis Infections

Seasonal trends are a notable feature of pseudotuberculosis epidemiology, with cases peaking during autumn and winter [20,21,58,76,77,79]. At the Wilhelma Zoo and the Opel Zoo, two-thirds of the cases occurred in colder months (*p* = 0.0023). Similarly, in the Antwerp Zoo, *Y. pseudotuberculosis* was primarily isolated during winter over a period of five years [90], and in two zoos in the UK, 83% of 24 cases occurred in winter [21]. In the Ramat Gan Zoo (Israel), 15 animals died over two winter months in 2012, coinciding with heavy rainfall and cold temperatures, but neither diet or population changes were noted nor outbreaks in other Israeli zoos [20]. In Belgium (2013), *Y. pseudotuberculosis* was significantly more prevalent in winter (26.9%) and spring (24.3%) than in summer (6.3%) [91]. In an Australian study focusing on *Yersinia* infections in weaned Merino sheep with diarrhea, *Y. pseudotuberculosis* was found only in winter, while *Y. enterocolitica* could be isolated throughout the whole year [60]. *Y. pseudotuberculosis* also showed seasonal dependence in free-ranging hares, with the highest incidence in November and December [92]. The seasonal occurrence of this pathogen in different species may be due to environmental contamination and pathogen multiplication, even under adverse conditions [60,93]. For zoo animals, increased susceptibility during winter may also be attributed to closer confinement in barns, elevated infectious pressure, and stress-induced immune suppression. Additionally, cold-stressed or parasite-infested animals are particularly vulnerable. Rodents, as latent carriers, invade barns for food and shelter, amplifying risks.

### 4.6. Study Limitations

Small mammals and wild birds found dead at the Opel Zoo were not included in the study, as were generally all fish, amphibians, and reptiles. Moreover, the effect of the autogenous vaccine based on isolates from the Wilhelma Zoo and the Opel Zoo was not investigated.

No immunological or environmental data were collected as part of the sampling at the Wilhelma Zoo and the Opel Zoo, with the exception of the division into urban and rural zoos and the consideration of season.

Further in-depth analyses of the *Y. pseudotuberculosis* isolates obtained in this study and other zoos using molecular techniques like whole-genome sequencing are pending and will be published elsewhere. Such investigations need to be conducted for molecular epidemiological studies and to assess the present of resistance and virulence genes. Further research on these issues would exceed the scope of this study.

## 5. Conclusions

In zoos, many different animal species live in a confined area. The present study provides a comprehensive overview of the prevalence of the zoonotic pathogen *Y. pseudotuberculosis* in numerous animal species and reveals that a number of these species are highly susceptible. In addition, there is the aspect of zoonosis, which means the risk of transmission of the pathogen to zookeepers, veterinarians, and visitors due to close contact to the zoo animals. This situation renders zoos as unique epidemiological units. However, this issue has not yet received sufficient attention, especially regarding the One Health approach.

Thus, in zoos, prophylactic and infection surveillance measures such as pest control, postmortem examinations of all deceased zoo animals, hygiene management—especially focusing on hand hygiene—and targeted vaccination strategies before the cold season play a decisive role in the control of *Y. pseudotuberculosis*.

## Figures and Tables

**Table 1:** Monitoring samples taken from animals at the Wilhelma Zoo and the Opel Zoo between June 2023 and May 2024.

Material	No. Samples (Wilhelma)	Positive	No. Samples (Opel Zoo)	Positive
Fecal samples (sick animals)	17	-	20	-
Fecal samples (‘hotspot species’)	108	-	61	1
Fecal samples (clinically healthy animals with increased risk of infection)	29	-	60	1
Necropsies (zoo animals)	148	2	18	3
Carcasses (wild small mammals and birds found dead on the grounds of Wilhelma)	311	6	-	-
Total	613	8	159	5

**Table 2:** Information on rodent species (R) year, month, gross pathological findings, matrices from which *Yersinia pseudotuberculosis* was isolated, and the zoo of origin.

Case No.	Species	Year	Month	Gross Pathological Findings	Source of *Y. pseudotuberculosis* Isolated	Zoo
R1	Brown rat *Rattus norvegicus*	2024	January	No organ abnormalities (pest control)	Liver, intestine	Stuttgart
R2	Brown rat *Rattus norvegicus*	2024	February	No organ abnormalities (pest control)	Liver, intestine	Stuttgart
R3	Brown rat *Rattus norvegicus*	2024	March	No organ abnormalities (pest control)	Liver, intestine	Stuttgart
R4	Brown rat *Rattus norvegicus*	2024	March	No organ abnormalities (pest control)	Liver, intestine	Stuttgart
R5	Capybara *Hydrochoerus hydrochaeris*	2007	n.a.^1^	n.a. ^1^	n.a. ^1^	Stuttgart
R6	Capybara *Hydrochoerus hydrochaeris*	2019	December	Multifocal necrotizing pneumonia, splenitis, hepatitis, and hyperplasia of mesenteric lymph nodes	Liver, spleen, lungs, kidney, intestine, lymph nodes	Schwerin
R7	Capybara *Hydrochoerus hydrochaeris*	2020	January	Swollen liver and spleen, liver with blunt margins	Liver, spleen, lungs, heart	Schwerin
R8	Capybara *Hydrochoerus hydrochaeris*	2020	January	Enteritis and necrotizing hepatitis	Liver, spleen, lungs, kidney, heart	Schwerin
R9	Chacoan mara *Dolichotis salinicola*	2019	April	High-grade purulent-necrotizing hepatitis and splenitis with bacterial foci	Liver, spleen, kidney	Wuppertal
R10	Chacoan mara *Dolichotis salinicola*	2023	March	High-grade necrotizing hepatitis, splenitis, enteritis, and pneumonia	Liver, spleen, lungs, kidney, intestine	Wuppertal
R11	Eurasian red squirrel *Sciurus vulgaris*	2024	January	No organ abnormalities	Liver, intestine	Stuttgart
R12	Guinea pig *Cavia porcellus*	2008	n.a. ^1^	High-grade purulent-necrotizing hepatitis, splenitis, mesenteric lymphadenitis, and pneumonia	Spleen	Berlin
R13	Guinea pig *Cavia porcellus*	2015	February	High-grade purulent-necrotizing hepatitis, splenitis, mesenteric lymphadenitis, and arthritis	Lymph nodes	Zurich
R14	Indian crested porcupine *Hystrix indica*	2023	March	Epiphora	Conjunctiva	Frankfurt
R15	Patagonian mara *Dolichotis patagonum*	2015	January	High-grade purulent-necrotizing hepatitis, nephritis, mesenteric lymphadenitis, and pneumonia	Liver, lungs, kidney, lymph nodes	Wuppertal
R16	Patagonian mara *Dolichotis patagonum*	2015	January	High-grade purulent-necrotizing hepatitis, nephritis, splenitis, and pneumonia	Liver, spleen, lungs, kidney	Wuppertal
R17	Patagonian mara *Dolichotis patagonum*	2017	August	Necrotizing multifocal hepatitis, mesenteric lymphadenitis, splenic hyperplasia, parasitosis with gastrointestinal nematodes	Liver, spleen, lungs, kidney, lymph nodes	Wuppertal
R18	Patagonian mara *Dolichotis patagonum*	2017	December	High-grade purulent-necrotizing hepatitis and splenitis, parasitosis with gastrointestinal nematodes and coccidia	Liver, spleen	Wuppertal
R19	Patagonian mara *Dolichotis patagonum*	2017	April	Abscess on shoulder, enteritis	Abscess	Wuppertal
R20	Patagonian mara *Dolichotis patagonum*	2017	April	Abscesses in liver, spleen, and pelvis	Liver, abscess in pelvis	Wuppertal
R21	Patagonian mara *Dolichotis patagonum*	2017	April	Necrotizing hepatitis, mesenteric lymphadenitis, spleen hyperplasia, high-grade purulent arthritis, and dermatitis on the hind limbs	Liver, spleen, lungs, kidney, intestine, arthritis	Wuppertal
R22	Patagonian mara *Dolichotis patagonum*	2020	October	Necrotizing hepatitis, mesenteric lymphadenitis, enteritis, spleen hyperplasia, and hyperemia	Liver, spleen, lungs, kidney, lymph nodes	Wuppertal
R23	Patagonian mara *Dolichotis patagonum*	2020	December	High-grade purulent-necrotizing pneumonia, hepatitis, splenitis, nephritis, lymphadenitis, parasitosis with coccidia	Liver, spleen, lungs, kidney, intestine	Wuppertal

Berlin: Tierpark Berlin; Frankfurt: Frankfurt Zoo; Schwerin: Schwerin Zoo; Stuttgart: Zoological-Botanical Garden Stuttgart Wilhelma; Wuppertal: Wuppertal Zoo; Zurich: Institute for Veterinary Pathology (IVP), University of Zurich; ^1^ n.a.: not available.

**Table 3:** Information on primate species (P), year, month, gross pathological findings, and matrices from which *Yersinia pseudotuberculosis* was isolated and the zoo of origin.

Case No.	Species	Year	Month	Gross Pathological Findings	Source of *Y. pseudotuberculosis* Isolated	Zoo
P1	Black-capped squirrel monkey *Saimiri sciureus*	2019	December	Necrotizing multifocal hepatitis, splenic hyperplasia with miliary-necrotizing inflammation, intestine with diphtheroid-necrotizing plaques	Liver, spleen, lungs, intestine	Stuttgart
P2	Black-capped squirrel monkey *Saimiri sciureus*	2024	January	Multiple granulomas in the liver parenchyma and spleen, necrotizing enteritis	Liver, spleen, intestine	Hodenhagen
P3	Black-capped squirrel monkey *Saimiri sciureus*	2024	January	Moderate multifocal necrotizing hepatitis, splenitis, and enteritis	Liver, spleen, intestine	Hodenhagen
P4	Black spider monkey *Ateles fusciceps rufiventris*	2018	January	High-grade multifocal necrotizing inflammation of liver and spleen	Liver, spleen	Wuppertal
P5	Black spider monkey *Ateles fusciceps rufiventris*	2018	January	High-grade hyperplasia of spleen and mesenteric lymph nodes	Liver, spleen, kidney	Wuppertal
P6	Black spider monkey *Ateles fusciceps rufiventris*	2018	January	Feces	Feces	Wuppertal
P7	Bonobo *Pan paniscus*	2017	March	Lungs with miliary necroses, inflammatory cells, and bacterial foci; multifocal colliquation necrosis in liver and spleen	Liver, lungs, kidney	Stuttgart
P8	Bonobo *Pan paniscus*	2017	March	Feces	Feces	Stuttgart
P9	Brown spider monkey *Ateles hybridus*	2019	December	Spleen, liver, mesenteric lymph nodes with highly multifocal foci of necrosis with bacterial foci, lymphoplasmacellular necrotizing enteritis	Liver, spleen, intestine	Munich
P10	Brown spider monkey *Ateles hybridus*	2020	February	Multifocal necrotizing hepatitis and splenitis, necrotizing myelitis with evidence of bacterial foci, diffuse catarrhal enteritis	Spleen	Neuwied
P11	Cotton-headed tamarin *Saguinus oedipus*	2021	April	High-grade multifocal necrotizing inflammation of liver	Liver	IVP Zurich
P12	De Brazza’s monkey *Cercopithecus neglectus*	2021	March	Necrotizing enteritis, splenitis, hepatitis, and pneumonia	Liver, lungs, spleen, kidney, intestine, heart	Donnersberg
P13	Emperor tamarin *Saguinus imperator*	2015	November	Multifocal purulent-necrotizing hepatitis, multifocal necrotizing splenitis; follicular hyperplasia and marked extramedullary hematopoiesis of the spleen	Liver, spleen, intestine	Neuwied
P14	Emperor tamarin *Saguinus imperator*	2019	December	Multiple granulomas in the liver parenchyma and spleen; generalized high-grade lymphatic hyperplasia	Liver, spleen, kidney, intestine	Private owner
P15	Geoffroy’s spider monkeys *Ateles geoffroyi*	2022	February	High-grade multifocal necrotizing inflammation of liver, liver capsular fibrosis, renal cysts, moderate non-suppurative nephritis	Liver	Karlsruhe
P16	Goeldi‘s marmoset *Callimico goeldii*	2022	December	Multifocal pyogranulomatous hepatitis, diphtheroid-necrotizing enterocolitis with lymph follicle proliferation, moderate splenomegaly	Liver, spleen	Stuttgart
P17	Patas monkey *Erythrocebus patas*	2019	November	Hyperplasia of spleen with colliquation necrosis, hepatitis with histiocytic infiltration, pyogranulomatous enteritis and necrotizing mesenteric lymphadenitis	Liver, spleen	Hamm
P18	Roloway monkey *Cercopithecus roloway*	2023	January	Necrotic foci in liver and spleen, granulomatous enteritis, splenomegaly, hyperplasia of mesenteric lymph nodes	Liver, spleen, lungs, kidney, intestine	Duisburg

Berlin: Tierpark Berlin; Donnersberg: Tierpark Donnersberg; Duisburg: Duisburg Zoo; Hamm: Tierpark Hamm; Hodenhagen: Serengeti Park; Karlsruhe: Karlsruhe Zoo; Munich: Tierpark Hellabrunn; Neuwied: Neuwied Zoo; Private: Private keeping in Lower Saxony; Stuttgart: Zoological-Botanical Garden Stuttgart Wilhelma; Wuppertal: Wuppertal Zoo; IVP Zurich: Institute for Veterinary Pathology, University of Zurich.

**Table 5:** Information on the infected bird species (B), order, year, month, gross pathological findings, and matrices from which *Yersinia pseudotuberculosis* was isolated and the zoo of origin.

Case No.	Species	Order	Year	Month	Gross Pathological Findings	Source of *Y. pseudotuberculosis*	Zoo
B1	Black grouse *Lyrurus tetrix*	Galliformes	2013	December	Liver and spleen highly enlarged with heterophilic infiltrates, lungs with heterophilic infiltrates and bacteria	Feces, intestine	Walsrode
B2	Black-naped Oriole *Oriolus chinensis*	Passeriformes	2014	January	n.a. ^1^	Swab sample from organs	Walsrode
B3	Black-necked aracari *Pteroglossus aracari*	Piciformes	2013	February	Granulomatous hepatitis and pneumonia with miliary necroses and bacterial foci	Liver, lungs, heart	Walsrode
B4	Black-necked aracari *Pteroglossus aracari*	Piciformes	2021	February	Liver and spleen with highly multifocal necroses with bacterial foci, kidney and lungs with bacterial foci and inflammatory infiltrates	Intestine, heart, visceral cavity, blood	Walsrode
B5	Blue-faced honeyeater *Entomyzon cyanotis*	Passeriformes	2022	January	Catarrhal-hemorrhagic enteritis	Liver, lungs, intestine, blood	Heidelberg
B6	Capuchinbird *Perissocephalus tricolor*	Passeriformes	2014	January	Liver enlarged, multiple granulomas, fibrinous inflammation and hemosidero-phagocytosis in liver, lungs, spleen	Liver, lungs	Walsrode
B7	Channel-billed toucan *Ramphastos vitellinus*	Piciformes	2021	May	Liver, lungs, adrenal gland, kidney with highly multifocal necroses with bacterial foci and arteriosclerosis	Liver, lungs, blood	Walsrode
B8	Channel-billed toucan *Ramphastos vitellinus*	Piciformes	2017	December	Spleen, liver, kidney with highly multifocal heterophilic granulomas, inflammatory infiltrates, and bacterial foci	Liver, lungs, intestine, heart, kidney	Walsrode
B9	Chestnut-eared aracari *Pteroglossus castanotis*	Piciformes	2016	April	Fibrinous hepatitis, splenitis, enteritis, nephritis, and pneumonia with bacterial foci	Liver, lungs, intestine, heart	Walsrode
B10	Chestnut-eared aracari *Pteroglossus castanotis*	Piciformes	2021	May	Liver, spleen, lungs with high-grade multifocal necroses, bacterial foci, and moderate inflammatory infiltrates and granulomas	Liver, lungs	Walsrode
B11	Common redshank *Tringa totanus*	Scolopacidae	2021	January	Liver, lungs, kidneys with bacterial emboli in the vessels partly with necrosis, hepatomegaly, cachexia	Liver, lungs, heart	Stuttgart
B12	Eastern rosella *Platycercus eximius*	Psittaciformes	2023	November	Liver with multifocal necroses, bacterial foci, and inflammatory infiltrates; lungs and spleen with multifocal pyogranulomas and bacterial foci	Liver, lungs, intestine, heart	Stuttgart
B13	Golden-headed quetzal *Pharomachrus auriceps*	Trogoniformes	2017	December	Liver, lungs, adrenal gland, kidney with severe multifocal necroses; bacterial foci and inflammatory infiltrates; hepatomegaly, splenomegaly	Liver, lungs, heart, visceral cavity	Walsrode
B14	Golden-headed quetzal *Pharomachrus auriceps*	Trogoniformes	2022	November	Lungs with edema and bacterial foci, liver with mild necrosis, bacterial foci, and inflammatory infiltrates	Liver, lungs, heart, kidney	Walsrode
B15	Golden-headed quetzal *Pharomachrus auriceps*	Trogoniformes	2022	November	Liver with severe multifocal necroses with bacterial foci, lungs and kidneys with bacterial foci	Liver, lungs, intestine, heart	Walsrode
B16	Green Aracari *Pteroglossus viridis*	Piciformes	2008	n.a. ^1^	Liver and spleen with miliary necroses, moderate catarrhal enteritis, cachexia	Liver, intestine, spleen	Berlin
B17	Green Aracari *Pteroglossus viridis*	Piciformes	2008	n.a. ^1^	Severe pneumonia, moderate splenomegaly, severe hemorrhagic enteritis, cachexia	Lungs, spleen	Berlin
B18	Green Aracari *Pteroglossus viridis*	Piciformes	2014	February	n.a. ^1^	Liver, lungs	Walsrode
B19	Green Aracari *Pteroglossus viridis*	Piciformes	2021	February	n.a. ^1^	Visceral cavity	Walsrode
B20	King bird-of-paradise *Cicinnurus regius*	Passeriformes	2023	February	Liver, spleen, lungs, ovary, serosa with high-grade and small intestine and kidney with low-grade multifocal necroses with bacterial foci	Liver, lungs, ovaries, blood	Walsrode
B21	Lesser flamingo *Phoeniconaias minor*	Phoenicopteriformes	2024	January	Liver, serosa, ovary, with miliary necroses; ascites, cachexia	Liver, lungs, heart, kidney, ovaries	Kronberg
B22	Madagascar turtle dove *Streptopelia picturata*	Columbiformes	2008	n.a. ^1^	Multiple granulomas in the liver, hemorrhagic enteritis	Liver	Berlin
B23	Magpie Shrike *Lanius melanoleucus*	Passeriformes	2013	January	Liver and spleen severely enlarged with multifocal necroses and bacterial foci	Liver	Walsrode
B24	Metallic pigeon *Columba vitiensis*	Columbiformes	2014	January	Inflammation and multiple granulomas in the liver, fibrinous splenitis and pneumonia, hemorrhagic enteritis	Intestine	Walsrode
B25	Pale-winged trumpeter *Psophia leucoptera*	Gruiformes	2015	November	Liver, kidney, spleen with severe multifocal fibrinous inflammation with bacterial foci, pneumoconiosis	Liver, lungs, heart, visceral cavity	Walsrode
B26	Pompadour cotinga *Xipholena punice*	Passeriformes	2008	n.a. ^1^	Severe granulomatous hepatitis, splenitis, and nephritis	Liver, spleen, kidney	Berlin
B27	Raggiana Bird-of-paradise *Paradisaea raggiana*	Passeriformes	2021	February	Liver, lungs, spleen with severe multifocal necroses and inflammation with bacterial foci, kidney with bacterial foci	Liver, lungs, brain, blood, feces	Walsrode
B28	Raggiana Bird-of-paradise *Paradisaea raggiana*	Passeriformes	2021	September	n.a. ^1^	Feces, throat	Walsrode
B29	Red-and-yellow barbet *Trachyphonus erythrocephalus*	Piciformes	2014	March	n.a. ^1^	Liver, lungs	Walsrode
B30	Red-rumped parrot *Psephotus haematonotus*	Psittaciformes	2023	January	Miliary necrotizing hepatitis with bacterial foci, hemorrhagic-catarrhal enteritis, cachexia	Liver, lungs, intestine, heart	Stuttgart
B31	Red-rumped parrot *Psephotus haematonotus*	Psittaciformes	2023	November	Miliary granulomatous necrotizing hepatitis, splenitis and encephalitis, diffuse satellitosis, tubulonecrosis, glomerulo-proliferation and single granulomas in kidney	Liver, heart	Stuttgart
B32	Spotted dove *Spilopelia chinensis*	Columbiformes	2022	November	Multifocal pyogranulomatous pneumonia with bacterial foci	Liver, lungs, heart	Stuttgart
B33	Toco toucan *Ramphastos toco*	Piciformes	2020	December	Moderate hepatomegaly, severe splenomegaly, severe miliary necroses in liver and spleen, pneumonia	Liver, lungs, spleen, kidney, heart	Wuppertal
B34	Toco toucan *Ramphastos toco*	Piciformes	2013	January	Liver and spleen enlarged, pericardium clouded with fluid accumulation, lungs highly reddened with fluid accumulation	Liver, lungs, intestine, heart, blood	Walsrode
B35	Toco toucan *Ramphastos toco*	Piciformes	2018	January	Liver and spleen with highly multifocal necroses with bacterial foci, in intestine and lungs, bacterial foci, and inflammatory infiltrates	Liver	Walsrode
B36	Toco toucan *Ramphastos toco*	Piciformes	2021	February	n.a. ^1^	Liver, lungs, heart, visceral cavity	Walsrode
B37	Toco toucan *Ramphastos toco*	Piciformes	2023	February	Liver and spleen with high-grade multifocal necroses with bacterial foci, bacterial foci in the lungs	Liver, lungs, visceral cavity, blood	Walsrode
B38	Toco toucan *Ramphastos toco*	Piciformes	2023	February	Liver and spleen with high-grade multifocal necroses with bacterial foci and inflammatory infiltrates, bacterial foci in the lungs	Liver, lungs, blood	Walsrode
B39	White-crested turaco *Tauraco leucolophus*	Musophagiformes	2013	February	Granulomas, inflammatory infiltrates, and masses of bacteria in liver, spleen, heart valve, small intestine, and kidney	Liver, lungs, heart	Walsrode
B40	White-throated toucan *Ramphastos tucanus*	Piciformes	2013	March	n.a. ^1^	Liver, intestine, heart	Walsrode
B41	White-throated toucan *Ramphastos tucanus*	Piciformes	2012	March	Liver and spleen severely enlarged with multifocal necroses, villous fusion and atrophy of the intestine	Liver, intestine	Walsrode

Berlin: Tierpark Berlin; Heidelberg: Heidelberg Zoo; Kronberg: Opel Zoo Kronberg; Stuttgart: Zoological-Botanical Garden Stuttgart Wilhelma; Walsrode: World Bird Park Walsrode; Wuppertal: Wuppertal Zoo; ^1^ n.a.: not available.

**Table 6:** Antimicrobial resistance profiles of 270 *Yersinia pseudotuberculosis* isolates from zoos included in this study. Testing and evaluation of the results were performed according to the CLSI version VET01-SEd7E guidelines for agar diffusion test [27]. Sensitive (S), intermediate (I), and resistant (R).

Subst. Abbrev.	CTX	S	TE	IMI	AK	CHL	CN	NOR	MER	AMC	NAL	W	SXT	AMP	CIP	CAZ	FEP
S	267	268	247	269	270	269	266	270	266	262	269	269	269	264	269	265	267
I	0	1	1	1	0	0	4	0	1	0	1	0	0	0	0	1	2
R	3	1	22	0	0	1	0	0	3	8	0	1	1	6	1	4	1

Substance abbreviations (Subst. Abbrev.): AMC (amoxicillin/clavulanate), AK (amikacin), AMP (ampicillin), FEP (cefepime), CTX (cefotaxime), CAZ (ceftazidime), CHL (chloramphenicol), CIP (ciprofloxacin), CN (gentamicin), IMI (imipenem), MER (meropenem), NAL (nalidixic acid), NOR (norfloxacin), S (streptomycin), TE (tetracycline), W (trimethoprim), and SXT (trimethoprim/sulfamethoxazole).

## Data Availability

The original contributions presented in the study are included in the article, further inquiries can be directed to the corresponding author.
